# Transformation of foldable robotic hand to scissor-like shape for pinching based on human hand movement

**DOI:** 10.1038/s41598-023-46622-x

**Published:** 2023-11-06

**Authors:** Hidetoshi Ikeda, Takumi Saeki

**Affiliations:** 1 Department of Engineering, Niigata Institute of Technology, 1719 Fujihashi, Kashiwazaki-City, Niigata Prefecture, Japan; 2 Department of Mechanical Engineering, National Institute of Technology, Toyama College, 13 Hongo-Chou, Toyama-City, Japan

**Keywords:** Engineering, Mechanical engineering

## Abstract

Increasing the number of degrees of freedom for multi-finger robotic hands is necessary to achieve high performance. However, this increases structural complexity and the obtained improvement may be small. Humans change the shape of their hands by extending or bending the fingers to apply force to an object through contact with a wide surface or two or more fingers. In some cases, continuous finger movements are not necessary or some fingers do not make contact with the object. A robotic hand with a small number of degrees of freedom could effectively use its fingers to perform many tasks by properly arranging the fingers, increasing the movable range of joints, and utilizing the back and sides of the fingers. This paper proposes a hand system and conducts a theoretical analysis of the transformation of the hand shape into a scissor-like motion to handle a cylindrical object. It is found that the scissor-like motion is unsuitable for cylindrical objects that exceed a certain size. Experiments show the effectiveness of the proposed hand system. The correlation between the contact position of a finger with an object and the success ratio of pinching is demonstrated. Furthermore, a control system that can switch from pinching to grasping when the robot judges that pinching is difficult is developed and experimentally validated.

## Introduction

Multi-finger robotic hands have many applications. Many multi-finger hand mechanisms have been extensively researched. Lévesque et al.^1^ studied a robotic hand with two fingers and two axes and Reynaerts et al.^2^ studied a robotic hand with two fingers and five axes. Dollar et al.^3^ studied a robotic hand with four fingers. Mouri et al.^4,5^ studied a human-like robotic hand with five fingers. Human-like robotic hands have been studied for application as robotic prosthetic hands ^6–8^.

In general, a multi-finger hand is necessary to achieve advanced tasks. However, such hands have relatively high structural complexity and their improvement may be limited. Thus, it is desirable to realize a robotic hand with relatively few actuators. Numerous underactuated robotic hands or grippers that have few actuators for driving joints have been studied ^9–13^.

The fingers of a multi-finger hand need to be controlled. Because the inside of a finger is relatively narrow, robotic hands with a tendon drive mechanism have been extensively researched ^14^. Yang et al.^15^ studied a linkage-spring-tendon-integrated compliant anthropomorphic robotic hand. Santina et al.^16^ studied a tendon-drive robotic hand with underactuated robotic fingers; the hand was able to open the cap of a glass bottle and pour coffee. Savić et al. ^17^ designed a robotic hand with a tendon drive mechanism and torsional springs that can adapt to the form of an object and grasp it.

Soft robotic hands that can handle delicate objects have also been investigated. Wang et al.^18^ studied a robotic hand with four soft fingers for handling groceries. Chen et al. ^19^ studied a robotic hand with three soft fingers that can sort various types of fruit. Soft hands or grippers for handling delicate marine organisms have been researched ^20,21^. Robotic hands with soft fingers that are arranged similar to a human hand have been studied and used to sort ripe tomatoes ^22^.

A robotic hand that uses the jamming of a granular material instead of fingers has been studied ^23^. A robotic hand that uses a jamming transition and a tendon drive mechanism has also been studied ^24^. A gripper mechanism with many pins that can conform to the shape of an object has been developed ^25^.

Multi-finger hands with functions that human hands lack have been studied. Research has been conducted on a hand that has two fingers and a sliding mechanism for precise handling using the surface of fingers ^26^, a hand with a mechanism that allows four fingers to fully rotate ^27^, and a hand with a ball mechanism at the tip of each finger ^28^. A review of robotic hands was reported by Bicchi ^29^.

Humans bend their fingers and change the shape of their hands to perform various tasks. Studies have been conducted on the taxonomy of the grasp type of human hands^30–33^.

The present authors previously designed a robotic hand that has a foldable mechanism and a wide movable range of joints and can use the back and sides of the fingers^34,35^. The hand has six degrees of freedom [DOFs) and can perform various tasks, such as placing, grasping, pinching, pulling, and picking up an object and turning the pages of a book. This paper reports the details of the hardware, system configuration, strategy for pinching using the hand, theoretical analysis of pinching a cylindrical object, and experimental results.

## Results

### Overview and design of robotic hand

In general, a robotic hand with many fingers can execute advanced tasks. However, the complexity of the required mechanism increases with the number of fingers and control becomes difficult. Therefore, in many cases, the number of fingers or axes for a robotic hand is decided by considering the task that the hand will perform. The robotic hand using the jamming phenomenon described above is capable of simple handling with a small number of degrees of freedom. On the contrary, the tools and systems in our living space are designed to be easily controlled by human hands, so technology based on multi-fingered robotic hands needs to be improved.

**Figure 1:**
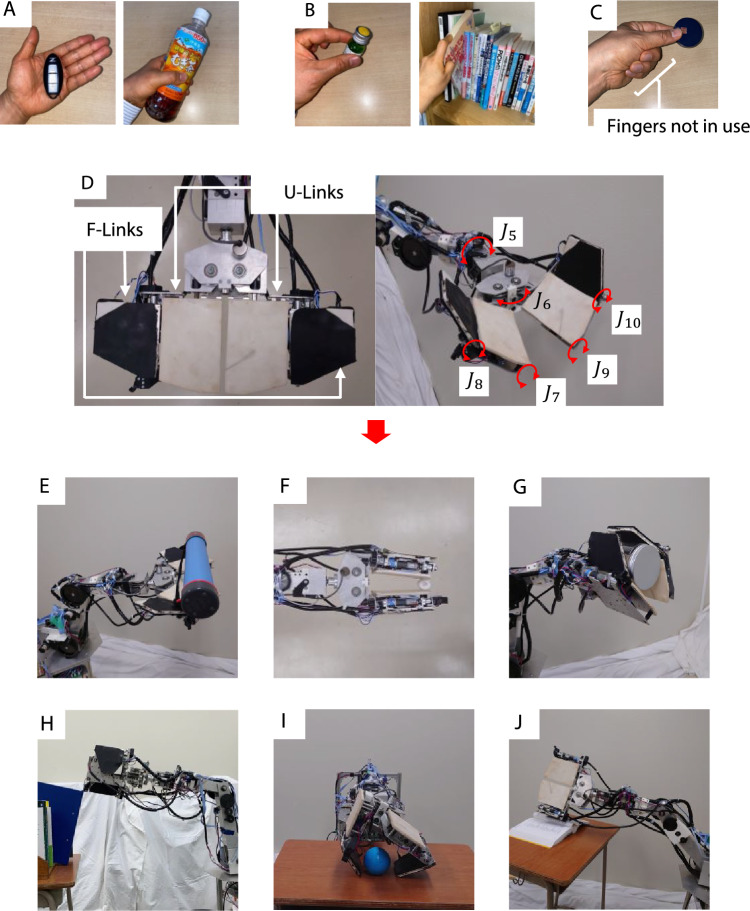
Motions of human hand and various tasks performed by robotic hand. (**A**) Human hand exerting a force in a plane against an object, (**B**) human hand exerting a force on an object at a point, (**C**) human fingers not acting on an object during handling, (**D**) 6-DOF robotic hand, JINZU (6 DOFs: ${J}_{5}$–${J}_{10}$, see “Materials and methods”) and robotic hand, (**E**) placing an object on its palm, (**F**) pinching, (**G**) grasping, (**H**) raking, (**I**) picking up an object, and (**J**) turning the pages of a book.

The human hand changes its shape by closing or extending the fingers and handles objects by exerting a force at one or several points with the fingertips or in a plane with the palm. It does not continuously drive all of the axes of the fingers; instead, it repeats small movements of the hand joints. For example, sequential movements of each joint of a finger are not always necessary when some fingers act as a surface, such as when simply placing an object on the palm of the hand or lifting and placing an object to be grasped (Fig. 1A). In such cases, the main aim is to secure a contact area between the hand and the object. A robotic hand with planar fingers for which the contact area is relatively large, even though the number of fingers is small, can execute this task.

Some tasks require an object to be handled at specific points, such as pinching a small object or pulling out a book from a bookshelf (Fig. 1B). When human fingers perform such tasks, in many cases, only a few fingers are used to handle the object; some fingers do not make contact with the object and thus do not dynamically act on the object (Fig. 1C).

A robotic hand with a small number of DOFs could effectively use its fingers and perform many tasks by properly arranging the fingers, increasing the movable range of joints, and utilizing the back and sides of the fingers.

In consideration of human hand movements and the application of a force to an object over a large contact area or at specific points, the authors designed a 6-DOF planar robotic hand called JINZU that can fold and transform (Fig. 1D) to handle an object. The hand has two plate fingers with a large movable joint range, allowing it to handle objects using not only the front surface of the fingers but also the back and sides of the fingers (U-Links and F-Links). The robotic hand can effectively utilize a few fingers and execute various tasks (Fig. 1E–J). The simplification of the robotic hand was also realized.

### Strategy for pinching object

**Figure 2:**
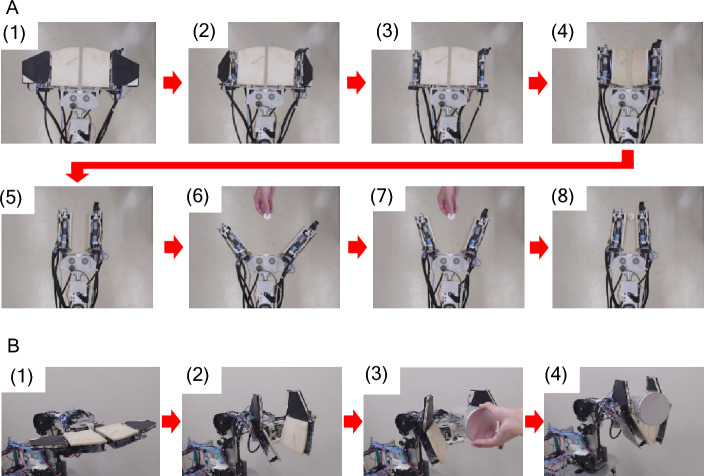
Process of (**A**) pinching and (**B**) grasping an object.

Figure 2A (1)–(8) shows the process of pinching, where both fingers are driven like a pair of scissors to pinch an object. In Fig. 2A (1)–(5), the robotic hand folds both fingers and turns them to a vertical orientation to perform the pinching motion. In Fig. 2A (6), the left and right fingers are opened. In Fig. 2A (7) and (8), the angle between the fingers is decreased to pinch the object. The pressure sensor attached to each finger detects the normal force against the object.

### Strategy for grasping object

Figure 2B (1)–(4) shows the process of grasping. In Fig. 2B (1), the left and right fingers are opened. In Fig. 2B (2), the robotic hand turns its fingers to a vertical orientation. The surfaces or back side of the fingers are moved to face toward the object using the wrist axis (${J}_{6}$, Fig. 1D). In Fig. 2B (3), the robotic hand increases the spacing between the right and left fingers to conform to the size of the object. In Fig. 2B (4), the robotic hand moves the surface of the finger closer to the object and the pressure sensor attached to each finger detects contact with the object. The fingers are closed to grasp the object. After the object is grasped, the manipulator lifts or places the object using the shoulder and elbow axes.

### Kinematics for robotic hand pinching cylindrical object

**Figure 3:**
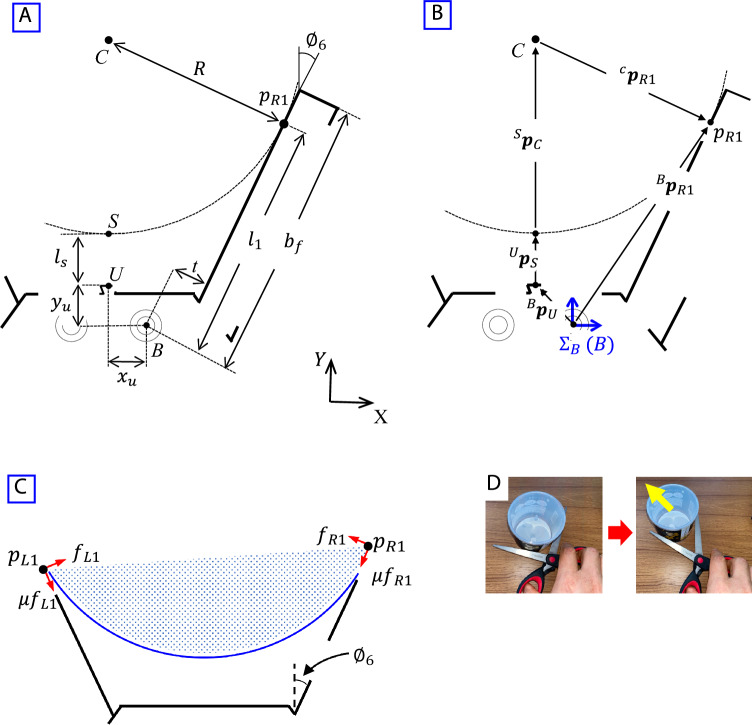
Model of pinching a cylindrical object and cutting motion of scissors. (**A**) Diagram of robotic hand, (**B**) position vectors in the system, (**C**) static model of robotic hand during pinching of a cylindrical object, and (**D**) cutting of a cylindrical object using pair of scissors.

Figure 3A–C show models of the robotic hand pinching a cylindrical object. When the robotic hand pinches an object, the fingers are driven like a pair of scissors. If the friction coefficient is relatively low, the scissor-like motion of the fingers sometimes pushes the object away because there are only two points of contact between the object and the fingers (this is similar to using a pair of scissors to cut a hard cylindrical object, with the object pushed forward, Fig. 3D). In this section, a theoretical analysis is conducted and the conditions required to pinch a cylindrical object are determined.

Figure 3A shows a model of the robotic hand pinching a cylindrical object and Fig. 3B shows the same model with the basic coordinate system of the hand denoted as ${\Sigma }_{B}$. The position vectors for the points in the system ${\Sigma }_{B}$ are shown in Table 1. Here, point *B* is the axis of the right finger (the right and left fingers are driven by one motor; see MATERIALS AND METHODS), point* U* is the position of the ultrasonic sensor used to detect the distance between hand and the object, point *S* is the closest position of the cylindrical object from the hand, point *C* is the center of the cylindrical object, and point ${p}_{R1}$ is the contact position between the right finger and the cylindrical object. ${b}_{f}$ is the length of the hand during pinching (width of finger, see Table 2 in MATERIALS AND METHODS) and ${l}_{s}$ is the distance between the finger base and an object, which is measured by the ultrasonic sensor. ${\phi }_{6}$ is the finger angle measured by the encoder on the hand (maximum finger angle is 50 deg). ${l}_{1}$ is the distance between the right finger (from the right axis) to the contact point with the object and $t$ is the distance between the center line of the finger and the finger surface.

**Table 1:** Parameters and position for pinching cylindrical object.

Position	Position vector from previous position	Parameter
Position of ultrasonic sensor (Point U)	$^{B}{{\varvec{p}}}_{U} : {[{-x}_{u} {y}_{u}]}^{T}$	${x}_{u}= 25 \mathrm{mm}, {y}_{u}= 25 \mathrm{mm}$
Closest distance to object from hand (Point S)	$^{U}{{\varvec{p}}}_{S} : {[0 { l}_{s}]}^{T}$	${l}_{s}$: Measured by ultrasonic sensor
Center of cylindrical object (Point C)	$^{S}{{\varvec{p}}}_{C} : {[0 R]}^{T}$	$R$: Radius of cylindrical object. It is detected based on finger angle (encoder) and closest distance to object (ultrasonic sensor)
Contact position between right finger and object (Point ${p}_{R1}$)	$^{C}{{\varvec{p}}}_{R1} :{[R\mathrm{cos}{\phi }_{6} -R\mathrm{sin}{\phi }_{6}]}^{T}$	${\phi }_{6}$: Measured from finger angle (encoder)
Contact position between right finger and object in system ${\Sigma }_{B}$	$$^{B}{{\varvec{p}}}_{R1} :\left[\begin{array}{c}{l}_{1}\mathrm{sin}{\phi }_{6}+t\mathrm{cos}{\phi }_{6}\\ {l}_{1}\mathrm{cos}{\phi }_{6}-t\mathrm{sin}{\phi }_{6}\end{array}\right]=\left[\begin{array}{cc}\mathrm{cos}{\phi }_{6}& \mathrm{sin}{\phi }_{6}\\ -\mathrm{sin}{\phi }_{6}& \mathrm{cos}{\phi }_{6}\end{array}\right]\left[\begin{array}{c}t\\ {l}_{1}\end{array}\right]$$	${l}_{1}$: Distance between right finger (from right axis) and contact point with object.$t$: distance between center line of finger and surface of finger ($t=1$ mm)

**Table 2:** Specifications of robot.

Overall length of wheeled mechanism	450 mm
Overall height of wheeled mechanism	270 mm
Radius of front wheels	15 mm
Radius of rear wheels	90 mm
Wheelbase	340 mm
Upper arm link of manipulator	300 mm
Forearm link of manipulator	220 mm
Thickness of wrist (${t}_{W}$)	78 mm
Width of robot base (${b}_{B}$)	84 mm
Thickness of robot base (${t}_{b}$)	86 mm
Length of U-link (${l}_{U}$)	132 mm
Length of F-link (${l}_{F}$)	104 mm
Thickness of fingers (${t}_{F}$)	30 mm
Width of fingers (${b}_{F}$)	164 mm

The position vector for the contact point between the surface of the right finger and the cylindrical object in the system ${\Sigma }_{B}$, $^{B}{{\varvec{p}}}_{R1}$, is expressed as

1 $$\begin{array}{*{20}c} {{}_{}^{B}{\varvec{p}}_{R1} = {}_{}^{B} {\varvec{p}}_{U} + {}_{}^{U} {\varvec{p}}_{S} + {}_{}^{S} {\varvec{p}}_{C} + {}_{}^{C} {\varvec{p}}_{R1} } \\ \end{array}$$

The following equation is obtained from Eq. (1) (Table 1):

2 $$\begin{array}{*{20}c} {\left[ {\begin{array}{*{20}c} {\cos \phi_{6} } & {\sin \phi_{6} } \\ { - \sin \phi_{6} } & {\cos \phi_{6} } \\ \end{array} } \right]\left[ {\begin{array}{*{20}c} t \\ {l_{1} } \\ \end{array} } \right] = \left[ {\begin{array}{*{20}c} { - x_{u} } \\ {y_{u} } \\ \end{array} } \right] + \left[ {\begin{array}{*{20}c} 0 \\ { l_{s} } \\ \end{array} } \right] + \left[ {\begin{array}{*{20}c} 0 \\ R \\ \end{array} } \right] + \left[ {\begin{array}{*{20}c} {R\cos \phi_{6} } \\ { - R\sin \phi_{6} } \\ \end{array} } \right]} \\ \end{array}$$

Thus,

3 $$\begin{array}{*{20}c} {\left[ {\begin{array}{*{20}c} t \\ {l_{1} } \\ \end{array} } \right] = \left[ {\begin{array}{*{20}c} { - x_{u} \cos \phi_{6} + R - y_{u} \sin \phi_{6} - l_{s} \sin \phi_{6} - R\sin \phi_{6} } \\ { - x_{u} \sin \phi_{6} + y_{u} \cos \phi_{6} + l_{s} \cos \phi_{6} + R\cos \phi_{6} } \\ \end{array} } \right]} \\ \end{array}$$

From Eq. (3),

4 $$\begin{array}{*{20}c} {t = - x_{u} \cos \phi_{6} + R - y_{u} \sin \phi_{6} - l_{s} \sin \phi_{6} - R\sin \phi_{6} } \\ \end{array}$$

5 $$\begin{array}{*{20}c} {l_{1} = - x_{u} \sin \phi_{6} + y_{u} \cos \phi_{6} + l_{s} \cos \phi_{6} + R\cos \phi_{6} } \\ \end{array}$$

${l}_{1}$ is the distance between the right finger (from the axis, point *B*) to the contact point (${p}_{R1}$) (Fig. 3A), which is obtained from Eqs. (4 and 5). ${b}_{f}$ (= 150 mm) is the finger length during the pinching motion. Therefore, ${l}_{1}$ is limited by ${b}_{f}$:

6 $$\begin{array}{*{20}c} {l_{1} = x_{u} + \frac{{\cos \phi_{6} }}{{1 - \sin \phi_{6} }}\left( {y_{u} + l_{s} + t} \right) \le b_{f} , \left( {0 \le \phi_{6} \le 50 deg} \right)} \\ \end{array}$$

Equation (6) shows that the robot can detect ${l}_{1}$(contact position between the fingers and a cylindrical object) using ${\phi }_{6}$ (finger angle) and ${l}_{s}$ (distance from the object to the hand).

### Static analysis of pinching cylindrical object

As described above, when a hand uses a scissor-like motion to pinch a cylindrical object, the object is sometimes pushed away (Fig. 3D) and the pinching fails. In this paper, the object is lightweight and it is assumed that the hand can lift it.

The robotic hand is assumed to slowly pinch an object and maintain its balance, which is achieved by an analysis that considers statics. This analysis is conducted to clarify the conditions required to avoid pushing the object away during the pinching motion.

In Fig. 3A,B, the left and right fingers are in contact with the cylindrical object at ${p}_{L1}$ and ${p}_{R1}$, respectively. ${f}_{L1}$ and ${f}_{R2}$ are the resistance forces from the left and right fingers at the contact points, respectively. Here, $\mu$ is the friction coefficient between the fingers and the object. The pinched object is affected by maximum friction forces $\mu {f}_{L1}$ and ${\mu f}_{R1}$ at the points of contact for the left and right fingers, respectively. It is assumed that there exists a limit state for the object not slipping. That is, the cylindrical object being pinched by the hand begins to slip when the coefficient of friction is lower than some limit.

The force vector at the contact points of the left U-link, ${{\varvec{f}}}_{L}$, is expressed as

7 $$\begin{array}{*{20}c} {{\varvec{f}}_{L} : \left[ {\begin{array}{*{20}c} {f_{L1} \cos \emptyset_{6} + \mu f_{L1} \sin \emptyset_{6} } \\ {f_{L1} \sin \emptyset_{6} - \mu f_{L1} \cos \emptyset_{6} } \\ \end{array} } \right]} \\ \end{array}$$

Similarly, the force vector at the contact points of the right U-link, ${{\varvec{f}}}_{R}$, is expressed as

8 $$\begin{array}{*{20}c} {{\varvec{f}}_{R} : \left[ {\begin{array}{*{20}c} { - f_{R1} \cos \emptyset_{6} - \mu f_{R1} \sin \emptyset_{6} } \\ {f_{R1} \sin \emptyset_{6} - \mu f_{R1} \cos \emptyset_{6} } \\ \end{array} } \right]} \\ \end{array}$$

From Eqs. (7 and 8), summing the total forces on the object exerted by the normal force and the friction force of the fingers for ${{\varvec{f}}}_{\Sigma B }\in {{\varvec{R}}}^{2}$ yields

9 $$\begin{array}{*{20}c} {{\varvec{f}}_{{{\Sigma }B{ }}} : \left[ {\begin{array}{*{20}c} {\left( {\cos \emptyset_{6} + \mu \sin \emptyset_{6} } \right)\left( {f_{L1} - f_{R1} } \right)} \\ {\left( {\sin \emptyset_{6} - \mu \cos \emptyset_{6} } \right)\left( {f_{L1} + f_{R1} } \right)} \\ \end{array} } \right] = \left[ {\begin{array}{*{20}c} 0 \\ 0 \\ \end{array} } \right]} \\ \end{array}$$

From Eq. (9), the cylindrical object is not pushed forward and the hand is able to pinch it when $\mathrm{sin}{\varnothing }_{6}-\mu \mathrm{cos}{\varnothing }_{6}\le 0$. In this case,

10 $$\begin{array}{*{20}c} {\mu \ge \tan \emptyset_{6} } \\ \end{array}$$

In addition, from Eq. (6),

11 $$\begin{array}{*{20}c} {\tan \phi_{6} = \frac{1}{{\cos \phi_{6} }} - \frac{{y_{u} + l_{s} + t}}{{l_{1} - x_{u} }}} \\ \end{array}$$

Thus, from Eqs. (10 and 11),

12 $$\begin{array}{*{20}c} {\mu \ge \frac{1}{{\cos \phi_{6} }} - \frac{{y_{u} + l_{s} + t}}{{l_{1} - x_{u} }}, \left( {0 \le \phi_{6} \le 50 deg} \right)} \\ \end{array}$$

To pinch a cylindrical object, the hand needs to satisfy the conditions given in Eqs. (6 and 12).

### Combinations of finger angle and distance from hand to object for pinching

**Figure 4:**
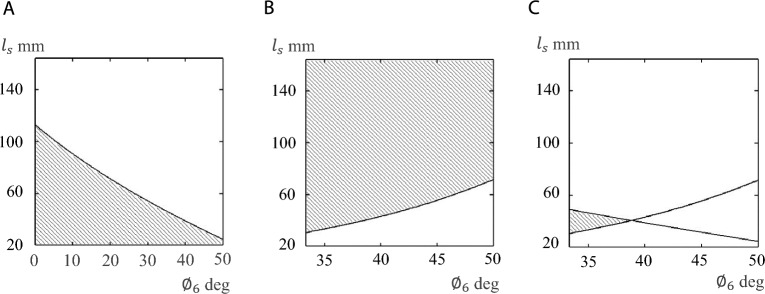
Relationship between finger angle, ${\varnothing }_{6}$, and distance to object, ${l}_{s}$, for pinching motion. Conditions required to (**A**) be in range of finger length and (**B**) avoid pushing object away. (**C**) Conditions required for pinching an object.

The shaded region in Fig. 4A shows the combinations of the finger angle, ${\phi }_{6}$, and the distance from the object to the hand, ${l}_{s}$, that allow the object to be within the range of the finger length, ${b}_{f}$, obtained from Eq. (6), where the ranges of the horizontal and vertical axes are respectively assumed to be $0\le {\phi }_{6}\le 50 \mathrm{deg}$ (maximum finger angle is 50 deg) and $20 \mathrm{mm}\le {l}_{s}\le 164 \mathrm{mm}$ (minimum value for the ultrasonic sensor to detect an object is 20 mm for finger length ${b}_{f}=164 \mathrm{mm}$). Selecting ${\phi }_{6}$ and ${l}_{s}$ in the shaded region in Fig. 4A guarantees that the contact position with the object will not exceed the finger length.

The shaded region in Fig. 4B shows the combinations of ${\phi }_{6}$ and ${l}_{s}$, obtained from Eq. (12), that make the robotic hand avoid pushing the object away. Here, the target object for pinching is a bucket (radius: 122.5 mm, friction coefficient $\mu$ = 0.70). It was assumed that the maximum contact position between the object and a finger is 85% of the finger length (i.e., 85% of the finger length is the maximum position for which the hand can stably pinch the bucket; see next subsection); in this case, the finger angle is ${\varnothing }_{6}$ = 33.31 deg (the calculation of the angle is shown in MATERIALS AND METHODS). The maximum angle of the finger is ${\varnothing }_{6}$ = 50 deg, and thus the range of the horizontal axis in Fig. 4B is 33.31 deg ${\le \varnothing }_{6}\le$ 50 deg. Selecting ${\phi }_{6}$ and ${l}_{s}$ in the shaded region in Fig. 4B guarantees that the object (bucket) will not be pushed forward and that the robotic hand will be able to pinch.

The shaded region in Fig. 4C is the overall result obtained from Fig. 4A,B. It represents the combinations of ${\phi }_{6}$ and ${l}_{s}$ that theoretically allow the robotic hand to pinch a cylindrical object (bucket). Since the shaded region is not large, it is difficult for a scissor-type robotic hand to pinch a cylindrical object above a certain size.

### Basic experiment on pinching objects

**Figure 5:**
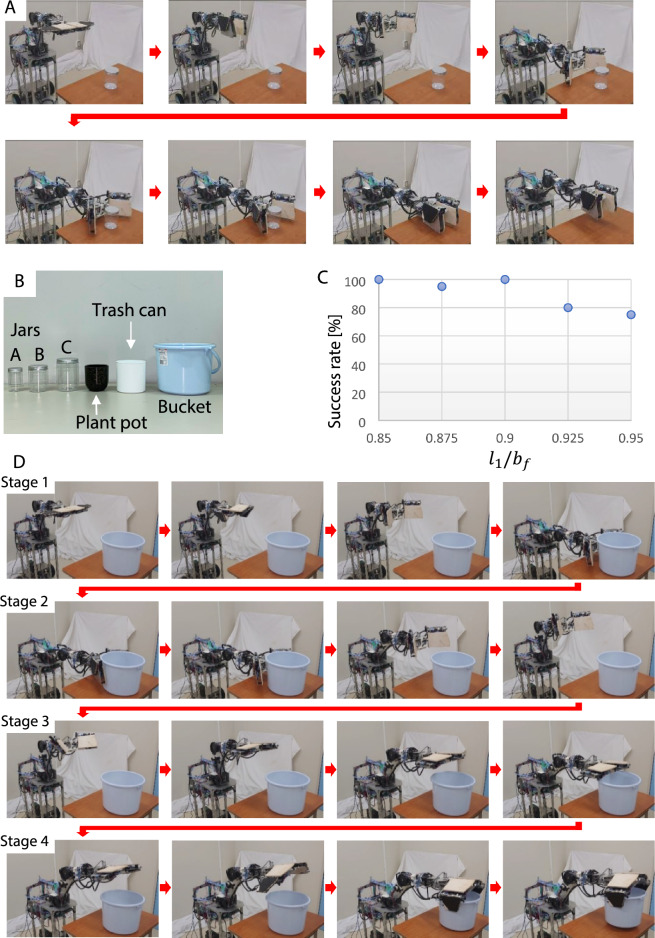
Experiment. (**A**) Sequence of pinching experiment, (**B**) objects used in pinching experiment, (**C**) success rate of pinching, and (**D**) sequence of experiment of switching from pinching to grasping.

First, an experiment with the robotic hand pinching a cylindrical object was conducted. The results show that the hand could detect, pinch, and lift the object (Fig. 5A). Here, the cylindrical object was a bottle (hereafter referred to as Bottle A; radius: 32.5 mm, mass: 28 g, material: PET, friction coefficient: 0.72, Fig. 5B).

Next, similar experiments were conducted. The objects to pinch were also bottles made of PET, but of a different size (radius: 42.5 mm, mass: 44 g, material: PET, friction coefficient: 0.72, hereafter referred to as Bottle B, and radius: 50 mm, mass: 75 g, material: PET, friction coefficient: 0.71, hereafter referred to as Bottle C, Fig. 5B). The hand was able to stably pinch and lift both objects.

Experiments using other objects were also conducted. The objects were a plant pot (radius: 55 mm, mass: 105 g, material: melamine resin, friction coefficient: 0.50, Fig. 5B) and a trash box (radius: 62.5 mm, mass: 110 g, material: polypropylene, friction coefficient: 0.68, Fig. 5B). The results show that the robotic hand was able to stably pinch and lift the objects.

### Correlation between the ratio of the finger length to the contact position and pinching success ratio

Additional experiments were conducted to clarify the correlation between the ratio of the finger length to the contact position with an object (${l}_{1}/{b}_{f}$, Fig. 3A) and the success rate of pinching a cylindrical object.

The robotic hand could stably pinch the objects described above because these objects were relatively small. The bucket was selected as the object to pinch and lift for the stability evaluation (diameter: 245 mm, mass: 266 g, material: polypropylene, friction coefficient: 0.70, Fig. 5B).

Before the experiments, many preliminary experiments were conducted using the bucket with ${l}_{1}/{b}_{f}$ values of less than 0.85 (${l}_{1}/{b}_{f}$ = 0.55, 0.65, and 0.75). Stable pinching by the robotic hand was confirmed. The robotic hand sometimes failed to pinch the bucket when the ${l}_{1}/{b}_{f}$ value was near 0.9. Figure 5C shows the results of an experiment conducted because the robot hand sometimes failed to pinch a large object when the contact position on the cylindrical object was close to the tip of the finger, resulting in it being decided that it was necessary to measure the success rate of pinching with the robot.

Thus, the experiments were conducted with ${l}_{1}/{b}_{f}$ = 0.85, 0.875, 0.9, 0.925, and 0.95 to clarify the success rate of pinching (Fig. 5C). The experiments were repeated 20 times each. The grasp position was set to 20 mm from the bottom of the bucket for lifting. In the experiments, success was judged when the hand was able to maintain the bucket in the air for 3 s without any part of the bucket touching the desk. Figure 5C shows the results of the experiment. As shown, the success rate gradually decreases after ${l}_{1}/{b}_{f}$ = 0.9. In the failure cases, the vibration during lifting was transmitted to the object, causing the bucket to tilt forward until the bottom touched the desk.

The reason for failure was lateral misalignment of the hand and cylindrical object positions, vibration caused by the movement of the wheel mechanism or manipulator before the pinching operation, or a combination of these factors.

### Conversion from pinching to grasping for handling object

The results of the theoretical analysis and the experiments indicate that the scissor-type robotic hand is not good at pinching a cylindrical object. Therefore, the authors developed a control system to convert the handling strategy from pinching to grasping when the robotic hand judges that pinching will be difficult.

The process of converting from pinching to grasping is divided into four stages (see stages 1–4, Fig. 5D). Stages 1 and 2 govern the process in which the robot determines the most suitable control motion to handle the object. Stages 3 and 4 govern the process in which the robotic hand transforms from a pinching motion to a grasping motion and handles the object.

*Stage 1*: The robot begins to pinch the object. The right and left fingers are folded and turned to a vertical orientation to pinch the object. The hand is opened. The robot lowers the hand and moves it forward toward the object based on the ultrasonic sensor on the hand. The robot stops at the position at which the hand can pinch the object.

*Stage 2*: The robot closes the hand. The limit switches on the fingers detect contact with the object. Using the measured values of the ultrasonic sensor, ${l}_{s}$, and encoder, ${\phi }_{6}$, the robot calculates the contact position between the hand and the object (${l}_{1}$, see Eq. 6). If ${l}_{1}/{b}_{f}$ is smaller than a set value, the robot continues pinching. Otherwise, the robot stops pinching and transforms the hand to grasp the object. The robot raises the hand position to avoid a collision between the hand and the object.

*Stage 3*: The robot transforms the hand shape to grasp the object and moves the hand toward the object. The robot moves the hand down and places it on the bucket. The robot moves backward to keep the horizontal position of the hand. The hand continues to move down. The force sensors on the fingers detect contact between the hand and the object. The height of the object is also detected.

*Stage 4*: The robot lifts the hand and moves forward to keep the horizontal position of the hand. The forearm links of the fingers (F-Links) are opened. The robot moves the hand down. The force sensors on the reverse side of the fingers detect contact between the hand and the object. Both fingers are closed. The hand grasps the object using the reverse side of the fingers and lifts the object.

The experiments on the conversion from pinching to grasping were conducted using a bucket as the target object (Fig. 5D). Here, the value of the control sequence for converting from pinching to grasping motion was set to ${l}_{1}/{b}_{f}$ = 0.6. The robotic hand began to pinch the object. It detected that the size of the bucket exceeded the set value. The hand changed from pinching to grasping motion and grasped and lifted the bucket.

## Discussion

A cylindrical object was used as the target object in this study. The kinematic analysis indicated that the contact position with the object can be calculated from the finger angle and the distance from the object to the hand. For pinching, the robotic hand performs a scissor-like motion with one DOF, in which the left and right fingers open and close. Therefore, depending on the finger angles and friction coefficients, the object might be pushed forward when it is pinched. A static analysis was conducted to clarify the conditions under which an object can be pinched. Numerical simulations based on the results of the above theoretical analyses were performed. The results show that a scissor-type hand is unsuitable for handling a cylindrical object. Thus, a control system was developed.

First, experiments of pinching cylindrical objects with different materials and sizes were conducted. The results confirmed that the robotic hand can stably pinch and lift objects. Next, experiments were conducted to determine the correlation between the contact position with an object and the success ratio of pinching. A bucket was used as the target object. The results show that the success ratio gradually decreased as the contact position moved closer to the tips of the fingers. From the results of simulations and experiments, a control system that changes the motion from pinching to grasping when the robot judges that pinching is difficult was developed. Experiments verified the effectiveness of the control system.

In this paper, the pinching motion of a robotic hand was evaluated. A theoretical analysis indicated that it is difficult to handle cylindrical objects with a scissor-type robotic hand. A foldable hand with a grasping strategy that can execute many tasks was realized. This robotic hand can use the sides and back of the fingers to perform various handling tasks with only 6 DOFs. Experimental results confirmed the effectiveness of the proposed hand. The correlation between the contact position of the finger with an object and the success ratio of pinching was shown. A control system that switches from pinching to grasping when the hand pinches a large object was developed. Experiments verified the effectiveness of the control system. This robotic hand is still under development and there is thus much room for improvement. For example, in the experiments on pinching objects conducted in this study, the authors assumed that the distance between the robot and the object to be grasped was not very great. However, in the case that the robot has to move a long distance to pinch an object, a physical system for adjusting hand position is needed. Therefore, the authors plan to analyze not only the hand but also the whole robot using dynamics to improve the system. On the contrary, it has been experimentally confirmed that the hand can perform many tasks, such as retrieving files from a bookshelf and turning the pages of a book. The most important thing to note is that this research realizes a robotic hand based on a new concept that focuses on the motion characteristics of the human hand which handles objects based on points and planes. Further, the effectiveness of the hand is reflected in that it has a greatly reduced number of degrees of freedom compared to conventional multi-fingered hands. Though already convinced that the robotic hand has various potential applications, the authors also believe in the necessity of validating them theoretically. We aim to realize such a validation and build a high-precision control system.

## Methods

This paper theoretically and experimentally examined the limits of pinching by the proposed hand system. The system was constructed based on hardware previously designed by the authors and some additional sensors or sensor systems.

### Robotic hand and wheeled mechanism

**Figure 6:**
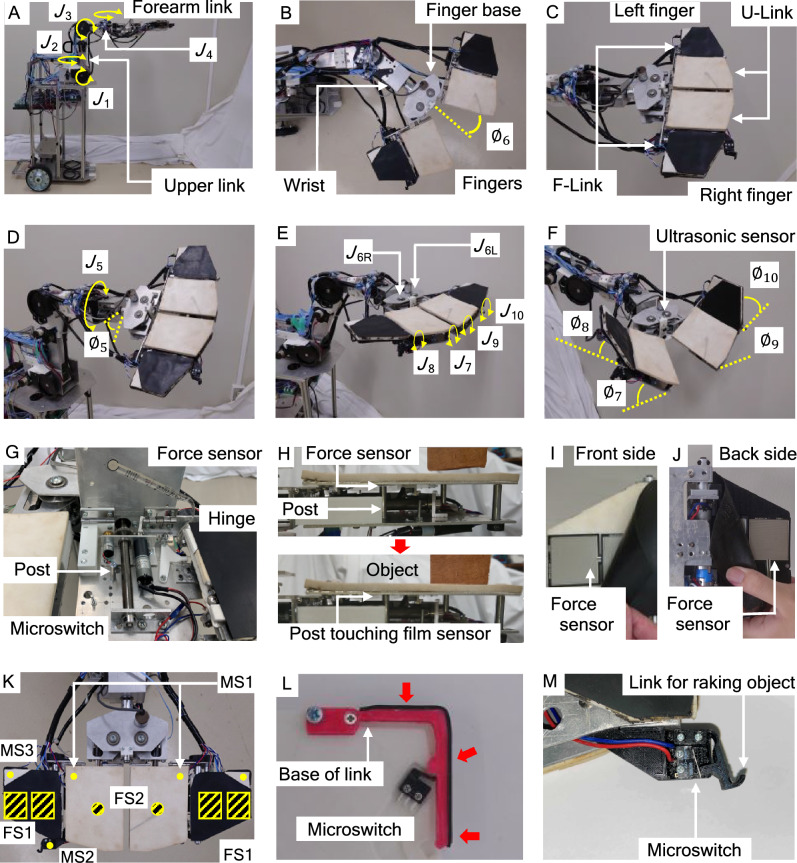
Mechanism and sensors of robotic hand. (**A**) Overview, (**B**) wrist and finger base (${\mathrm{\varnothing }}_{6}$: angle of finger), (**C**) forearm links (F-Links) and upper links (U-Links) of fingers, (**D**) angle in roll direction around *J*_5_ (${\mathrm{\varnothing }}_{6}$), (**E**) axes of finger base and fingers, (**F**) angles of finger axes (${\mathrm{\varnothing }}_{7}-{\mathrm{\varnothing }}_{10}$) and position of ultrasonic sensor on finger base, (**G**) force sensors inside U-Link, (**H**) action of U-Link for touching object, (**I**) force sensor on front side between two sponge rubber parts, (**J**) force sensor on back side between two sponge rubber parts, (**K**) arrangement of sensors (FS1 and FS2 are force sensors and MS1-MS3 are microswitches), (**L**) touch sensor system for raking an object designed in 3D-CAD, and (**M**) touch sensor system designed in 3D-CAD.

The foldable robotic hand, JINZU, and the wheeled mechanism were developed in our laboratory (Fig. 6A). The wheeled mechanism has two pairs of wheels, each of which consists of a left wheel and a right wheel. The front pair is casters and the rear pair is driving wheels. The movement mechanism uses left and right individually opposing drive systems, which are equipped with two motors (Tsukasa Electric Co., LTD., TG-85E-SU-47.9-KA, 24 V) and two encoders (AUTONICS. E30S4-100-3-N-5). The robotic hand is set on the forearm link of the manipulator, which is set on the body of the wheeled mechanism. This manipulator has shoulder axes (*J*_1_, *J*_2_, Fig. 6A) and elbow axes (*J*_3_,* J*_4_). There are motors and encoders on each axis (*J*_1_–*J*_3_, motor: Tsukasa Electric Co., LTD., TG-85E-KU-113-KA, 24 V, encoder: NIDEC COPAL ELECTRONICSRE12D-100-201-1. *J*_4_, motor: TG-101C-GU-581-KA, 24 V, encoder: RECW20D-25–201-1). The robotic hand has 6 DOFs and 7 joints. It consists of three parts, namely the fingers, finger base, and wrist (Fig. 8B). The fingers are right and left fingers, each of which has two links (U-Link and F-Link, Fig. 6C). The insides of the right and left U-Links have motors and encoders to control the fingers (*J*_7_–*J*_10_, motor: Tsukasa Electric Co., LTD., TG-85E-KU-113-KA, 24 V, encoder: NIDEC COPAL ELECTRONICSRE12D-100-201-1). The fingers make contact with objects. Sponge rubber parts (Misumi Corporation, PRGCW5, color: white, and Misumi Corporation, SGNS10-500-800, color: black, Fig. 6C) are glued on the surfaces of the fingers. The wrist has an axis,* J*_5_, that has a motor and an encoder (*J*_5_, motor: Tsukasa Electric Co., LTD., TG-101C-GU-581-KA, 24 V, encoder: NIDEC COPAL ELECTRONICS, RE30E-360-213-1). It is able to rotate the assembly that consists of the finger base and a finger (Fig. 6D) in the range $0\le {\varnothing }_{5}\le 180$ deg.

The robotic hand changes its shape to handle various objects by folding the fingers. The right and left fingers are attached to the finger base (Fig. 6B). The base has a motor and an encoder (*J*_6_, motor: Tsukasa Electric Co., LTD., TG-101C-GU-581-KA, 24 V, encoder: NIDEC COPAL ELECTRONICS, RE12A-100-100-1) and two joints (6R and 6L, Fig. 6E). One motor controls joints 6R and 6L and changes the finger angle, ${\varnothing }_{6}$ in the range $-10$ deg $\le {\varnothing }_{6}\le 50$ deg (when fingers are vertical). The angles of the fingers can be individually driven (Figs. 6E,F). The joint angles of the left and right U-Links (${\varnothing }_{7}$ and ${\varnothing }_{9}$) are $-90$ deg $\le {\varnothing }_{7}\le$ 90 deg and $-90$ deg ${\le \varnothing }_{9}\le 90$ deg, respectively, and those of F-Link (${\varnothing }_{8}$ and ${\varnothing }_{10}$) are − 180 deg $\le {\varnothing }_{8}\le 60$ deg and − 180 deg $\le {\varnothing }_{10}\le 60$ deg.

The force sensors (film sensors) (Tekscan Corp., A201, High 445 N, 0-–100 lb) and microswitch sensors (OMRON Corp., D2F-01L-D) are installed on the inside of the U-Links of the fingers (Fig. 6G). Each U-Link has posts inside it (Fig. 6H). When the hand makes contact with an object, the posts push each force sensor, which detects contact. Four force sensors (INTERLINK ELECTRONICS Inc., FSR406) are installed on the front of each F-Link (Fig. 6I,J). Sponge rubber parts are set on each sensor. Four sensors are also installed on the back of each F-Link. The arrangement of sensors is shown in Fig. 6K. Touch sensor systems for scooping or picking up an object (Fig. 6L) and raking an object (Fig. 6M) are also installed. The systems were designed in 3D-CAD and each has a microswitch (OMRON Corp., D2MQ-01L-D, D2MQ-4L-1). These systems were not used in this paper.

**Figure 7:**
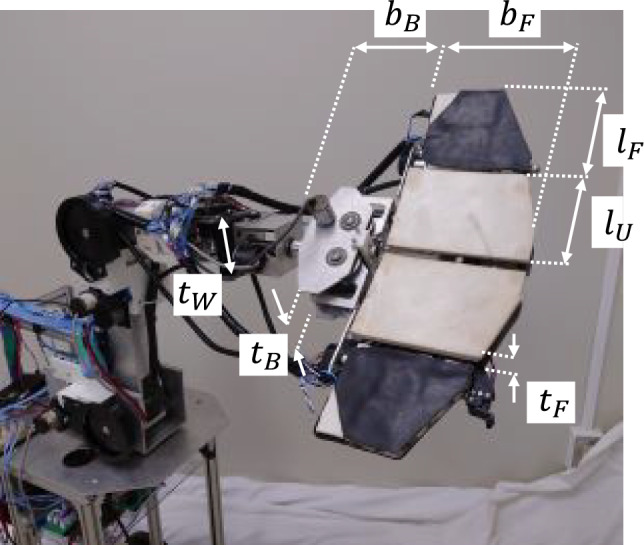
Schematic diagram of robotic hand mechanism.

Table 2 shows the specifications of the robot and Fig. 7 shows a schematic diagram of the robot.

### System configuration of robot

**Figure 8:**
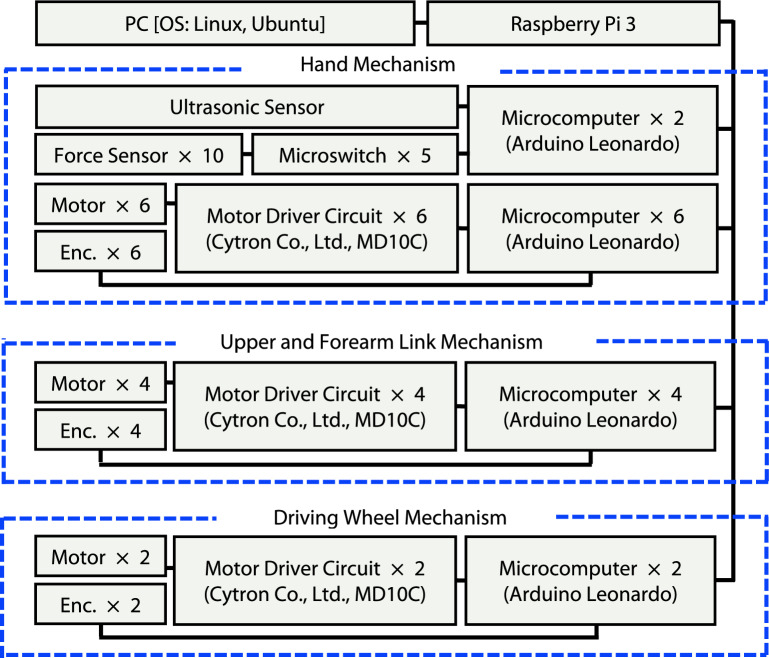
System configuration.

Figure 8 shows a configuration diagram for the system controls. The motors and encoders of the robotic hand (6 DOFs), the upper link and forearm link of the manipulator (4 DOFs), and the driving wheel system (2 DOFs) (total of 12 motors) are connected to each 12-motor driver circuit separately (Cytron Co., Ltd., MD10C). The rotary encoders permit the shaft position of these motors to be detected. The motor driver circuits are connected to each microcomputer (Arduino Holdings, Arduino Leonardo) to control the driving process. The ultrasonic sensor (Fig. 6F), microswitches and force sensors (Figs. 6G–M) are connected to two other microcomputers. A total of 14 microcomputers (Arduino Leonardo) are used as slave microcomputers. They are connected to a Raspberry Pi 3, which acts as the master microcomputer. The Raspberry Pi 3 is connected to a PC via LAN cables and connectors. The PC runs a Linux operating system (Ubuntu 22.10). The operating software on the master microcomputer (Raspberry Pi 3) was developed in C++. The operating software on the slave microcomputers (Arduino) was developed in the Arduino language. The control program was developed in Windows 10 and installed on the slave microcomputers. The robot system was controlled by command signals from the master microcomputer. The 14 slave microcomputers received the signals from the master microcomputer and controlled the motors. The microcomputers also received the values from the sensors and sent signals to the master microcomputer.

### Method of calculating ${\boldsymbol{\varnothing }}_{6}$ when hand pinches bucket in simulation

The minimum angle for pinching the bucket (${\varnothing }_{6}$) is 35.45 deg when *l*_1_ is equal to 0.85% of ${b}_{f}$ (see Fig. 4B). This is because, based on the results of the basic experiments (see RESULTS), it was determined that 85% of the finger length, ${l}_{1}$, is the maximum contact position (thus, minimum angle of finger) at which the bucket can be stably pinched. Thus, the simulations were performed on the bucket in the range 35.45 deg ≤ ${\varnothing }_{6}$ ≤ 50 deg (Fig. 4B,C). Here, 50 deg is the maximum angle of the hand mechanism and 35.45 deg was determined using Eq. (13). The derivation of the equation is as follows.

Solving Eq. (4) $\times \mathrm{cos}{\varnothing }_{6}+$ Eq. (5)$\times \mathrm{sin}{\varnothing }_{6}$ yields

$$\begin{array}{*{20}c} {l_{1} \sin \emptyset_{6} = - x_{u} + \left( {R - t} \right) \cos \emptyset_{6} } \\ \end{array}$$

Thus,

13 $$\begin{array}{*{20}c} {\cos \emptyset_{6} = \frac{{x_{u} \left( {R - t} \right) + l_{1} \sqrt {\left( {R - t} \right)^{2} - x_{u}^{2} + l_{1}^{2} } }}{{\left( {R - t} \right)^{2} + l_{1}^{2} }}} \\ \end{array}$$

### Supplementary Information

Supplementary Video 1.

## Data Availability

All data and materials are available in the paper and Supplementary Materials.
